# Effects of Maximal Effort Running on Special Agents’ Loaded and Unloaded Drop Jump Performance and Mechanics

**DOI:** 10.3390/ijerph181910090

**Published:** 2021-09-25

**Authors:** Justin J. Merrigan

**Affiliations:** Human Performance Innovation Center, Rockefeller Neuroscience Institute, West Virginia University, Morgantown, WV 26505, USA; justin.merrigan@hsc.wvu.edu

**Keywords:** occupational health, tactical athlete, landing error scoring system, reactive strength index, tactical personnel, force plates, military, law enforcement, neuromuscular fatigue

## Abstract

The purpose was to investigate the effect of load and fatigue on landing forces and mechanics. Thirteen Department of State special agents first completed drop jump testing, a maximal treadmill test, and another round of drop jump testing. During drop jump testing, agents performed 3 maximal effort drop jumps from 30 cm with body mass only (unloaded) or a 15 kg weight-vest (loaded). A force plate was used to collect force–time data, while two laptops were placed 3 m from the force plate from frontal and sagittal planes. Two-way analyses of variance were used to analyze the effect of load and fatigue on landing forces and Landing Error Scoring System (LESS) with alpha of *p* < 0.05. Dropping from 30 cm with 15 kg resulted in greater landing impulse, which was driven by increases in contact time. The loaded condition also resulted in lower jump height and reactive strength indexes. After the maximal graded treadmill test there were no further changes in drop jump ground reaction forces or performance. However, relative aerobic capacity was related to impulse changes following the treadmill test in unloaded (R^2^ = 0.41; *p* = 0.018) and loaded conditions (R^2^ = 0.32; *p* = 0.044). External loads of 15 kg increased impulse and contact time and resultantly decreased drop jump height and reactive strength indexes. It is encouraged that training protocols be aimed to concomitantly improve aerobic capacity and lower body power. Plyometric training with progressive overloading using external loads may be helpful, but further research is warranted.

## 1. Introduction

Special agents endure intensive physical training to best prepare them for their demanding and unpredictable occupational tasks. During training, 35% of male and 42% of female agents experience one or more injuries, which are most likely to occur at the knees and thighs [1]. These musculoskeletal injuries lead to health consequences for the individual and undue government funding and resources [2]. Risk factors for injury occurrences include traits of physical prowess, such as aerobic capacity [1,3], neuromuscular capabilities (i.e., strength and power) [4], and movement mechanics [3,5]. Injury risk is also heighted during tasks requiring load carriage [6] and/or tasks that acutely induce high levels of fatigue [7,8]. Therefore, strategies have been employed to evaluate physical capabilities in tactical populations, particularly while under external loads or during periods of heightened fatigue [9].

Vertical jump testing is one method used to identify levels of fatigue during sustained operations training in the military [10]. To further evaluate neuromuscular performance capabilities under load and fatigue, jump testing is being conducting on force plates in tactical populations [9,11,12]. The benefit of examining a movement’s (e.g., countermovement jump’s) force–time characteristics, is the additional data pertaining to the forces and movement strategies required to execute the movement. For example, the individual may adopt different movement strategies (e.g., shorter contraction times, deeper or shallower countermovement depths) in attempt to attain the same maximal effort jump heights as their last testing session [13]. Although these data are useful for identifying forces generated by or acting on the body and general movement mechanics, further data collection methods are necessary to ascertain movement patterns regarding specific joints.

To identify these biomechanical movement patterns, expensive laboratory motion capture equipment is often used [14]. However, when traditional laboratory equipment is not permitted, practitioners may consider using subjective field tests, such as the Landing Error Scoring System (LESS) [14]. The LESS is a clinical tool for assessing potential errors in movement mechanics (i.e., knee valgus) during landing and jumping tasks through visual inspection of front and side view recordings [15]. The resultant score is a summation of “errors” identified throughout various stages of the movement. Higher scores allude to poorer mechanical movement patterns, which may occur due to fatigue [16] and are associated with knee injuries in tactical populations [5]. Thus, these tools may be useful to help evaluate injury risk factors during jumping and landing tasks.

For example, external loads required of tactical personnel may lower jumping performance [14,17] and alter movement strategies and forces imposed on the individual when landing [9,14,18,19]. These biomechanical analyses informing the effects of external loading may preface the negative influence of external loading on injury risk [20] and performance of high-intensity tactically related duties (e.g., combative movements) [21,22,23]. Similarly, movement mechanics, such as LESS, have been linked with injury risk [3,5] and are often impaired due to acute bouts of fatiguing tasks [16,24,25]. Peak landing forces have also been increased, alongside incidences of stress fractures, during fatiguing tasks [26]. However, some have failed to find altered vertical ground reaction forces (vGRFs) from exercise induced fatigue [27]. Yet, despite no changes in jump height or peak vGRF following a fatiguing bout of running, knee valgus increased [24]. Thus, a combined analysis of the vGRF and movement mechanics may better inform the cumulative effects of running induced fatigue and external loading, which is necessary to investigate as running prior to landing tasks may impair mechanics and performance in loaded conditions [19].

Physical prowess may also reduce the negative effects of external loading or fatigue on movement patterns. For example, stronger individuals may note less reductions in drop jump performance [14]. Likewise, individual’s that are more aerobically fit require a lower working capacity to achieve the same running outcome as individual’s that are less aerobically fit [6]. Investigating the relationship between aerobic capacity and alterations in movement patterns from fatigue may, in part, help to explain the potential mediation between aerobic capacity as a risk factor for injury [1] and the common occurrence of injuries during load carriage tasks [6]. This may be particularly pertinent to investigate in the Department of State Diplomatic Mobile Security Deployment (MSD) Special Agents who endure intense training to prepare them to operate in high-threat environments with little outside support. These agents are often deployed to global hotspots to be readily available for quick responses to protect U.S. federal government officials from kidnapping and terrorist threats and to protect and evacuate U.S. citizens out of crisis areas. Despite some aspects of the day-to-day operations of MSD special agents being considered sedentary (i.e., screening visitors), their training and operations often involve advanced and precise firearm handling and tactics, close quarters combat, counter-terror tactics, off-road and/or high-speed vehicle operations, advanced navigation, first-aid, and survival capabilities under fatigued and loaded conditions. Thus, the purpose of this study was to investigate the effect of external loading and short bouts of maximal effort running on landing forces and mechanics in MSD agents and whether their aerobic fitness levels would influence the effects of running on drop jump results.

## 2. Materials and Methods

### 2.1. Subjects

According to average effects of training induced fatigue on jump performance from prior research [11], large effects were anticipated revealing an a priori minimum sample size estimate of 12 for the current study. Thirteen Department of State MSD Special Agents (age, 37 ± 5 years; body mass, 71.67 ± 3.81 kg; height, 202.11 ± 28.71 cm; VO_2max_, 4.18 ± 0.63 L∙min^−1^; relative VO_2max_, 45.50 ± 4.10 mL∙kg∙^−1^min∙^−1^) participated. All agents had at least 2 years of consistent physical training and were considered healthy in accordance with the physical activity readiness questionnaire (PAR-Q). Agents were asked to refrain from activities that may fatigue musculature and inhibit their ability to perform the current tasks for 48 h prior to arriving to the laboratory (ex. resistance training, high volume or intensity running or occupational tasks). Participants were also asked to adhere to normal sleeping and eating habits and avoid alcohol, tobacco, caffeine, and other ergogenic aids/supplements for at least 3 h before testing.

### 2.2. Design

To determine the effects of short bursts of maximal running on neuromuscular performance and mechanics, special agents completed drop jump testing before and after a maximal treadmill test, separated by 2 min of rest. Prior to testing, agents completed a short dynamic warm-up (5 min cycle and 5 min lower body dynamic stretching) and were familiarized with the drop jump protocols. All testing procedures occurred at approximately the same time of day (1000–1300) under the supervision of certified strength and conditioning specialists (NSCA CSCS).

### 2.3. Maximal Graded Treadmill Testing

During treadmill (ELG, Woodway, Waukesha, WI, USA) protocols, inspired and expired gases were transferred through a two-way valve into a gas analyzer (ParvoMedics TrueOne 2400 Metabolic Cart) to assess aerobic capacity (VO_2max_). Throughout the test heart rate was assessed using a chest strap device (PolarH7, Kempele, Finland). The first and second stages were a warm-up at 5.0 km∙hour^−1^ with 0% grade and 6.5–8.0 km∙hour^−1^ at a 5.2% incline, respectively. The remainder of the maximal treadmill test was performed at 5.2% incline. Speed began at their 2-mile run pace and increased after each one-minute stage by 1.0 km∙hour^−1^ until volitional fatigue. All tests were completed within 7-10 min and considered true maximal tests based on previous criteria, described elsewhere including: plateau of oxygen uptake despite an increase in workload, respiratory exchange ratio above 1.10, achieving 90% of their age estimated max heart rate (206.9 − 0.67×ge), rating of perceived exertion greater than or equal to 18 (from Borg-scale of 6-20); and a venous blood lactate >8 mM [28]. The blood lactate was collected from a fingertip, cleaned by an alcohol swab, through a small incision from a lancet (Tenderlett; Accriva Diagnostics; San Diego, CA). The initial blood sample was wiped away with medical gauze, and the subsequent drop was used for analysis. A collecting strip (Lactate Plus meter test strips; Nova Biomedical, Waltham, MA, USA) was inserted into a portable lactate analyzer (Lactate Plus meter; Nova Biomedical), which was calibrated with a control solution (Lactate plus control solution level 2; Nova Biomedical) according to factory guidelines.

### 2.4. Drop Jump Testing and Analysis

During drop jump testing, agents performed 3 maximal effort jumps from a 30 cm box without and with a 15 kg weight-vest, in random order using a counterbalanced design (7 participants begin with unloaded condition and 6 with loaded condition). Rest between jumps was 30-s while rest between conditions was 1 min. Agents were instructed to step off, not walk or jump off, the box and immediately perform a maximal effort countermovement jump with little ground contact.

Force–time data were collected from a portable force plate (AccuPower; AMTI, Watertown, MA, USA) via a custom-built interface box with an analog-to-digital card (NI cDAQ-9174; National Instruments, Austin, TX, USA) at 1600 Hz and analyzed using Matlab (version 7.12, MathWorks, R2011a, Natick, MA, USA). The landing phase was identified from ground contact, when forces were >5 standard deviations above the one-second quite weighing phase average, to takeoff, when forces were <5 standard deviations of the quite weighing phase. The following force–time metrics were calculated during the entire landing contact duration and used in analyses: peak vGRF, maximal vertical ground reaction force; impulse, area under the curve; rate of force development, change in vGRF from contact to 20 milliseconds after contact divided by 20 milliseconds; contact time, duration from contact to takeoff; flight time, time from takeoff to second ground contact; jump height, 0.5 × 9.8 × (flight time/2)^2^; reactive strength index, flight time divided by contact time.

Videos, for LESS, were taken from two laptops (ThinkPad, Lenovo, Morrisville, NC, USA) with the same video recording capabilities and quality (resolution, 720p; frame rate, 30 fps), placed 3 m from the participant in frontal and sagittal planes. Drop jumps were analyzed using computer software (QuickTime; Apple, Inc, Cupertino, CA, USA) from ground contact, frame immediately prior to complete foot contact, to maximal knee flexion, using a scoring sheet described elsewhere [15]. The average total LESS score for each condition was used for analysis with a higher score indicating more landing errors.

### 2.5. Statistical Analysis

Data were considered normally distributed according to Shapiro–Wilks and visual inspection of histograms. Reliability of was calculated across the three trials for each timepoint and condition using the coefficient of variation with a threshold of >10% determining an unreliable metric. Two-way analyses of variance were used to analyze the effect of load and fatigue on landing vGRFs and LESS. The association between relative VO_2max_ and the decrease in drop jump performance was assessed via linear regression analyses. Cohen’s d effect sizes were calculated with corresponding 95% confidence intervals with the following determinants: <0.2, negligible; 0.20–0.49, small; 0.50–0.79, moderate; >0.80, large. Analyses were conducted using R, version 3.6.2 [29] with *p* < 0.05.

## 3. Results

**Figure 1:**
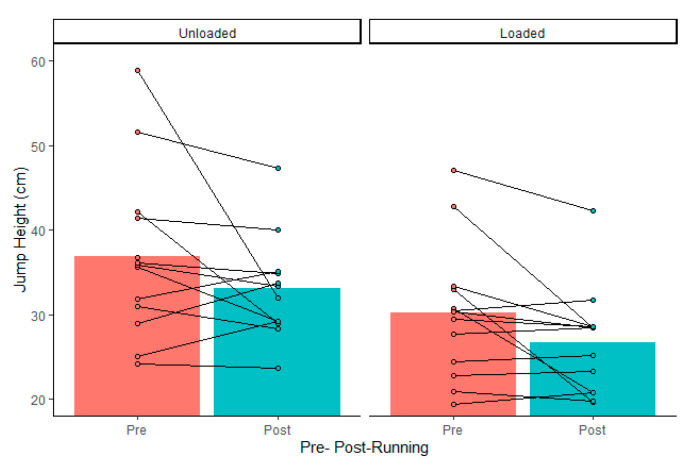
Group average (gray bars) and individual data (dots and lines) for drop jump height before (Pre) and after (Post) the maximal graded treadmill test in unloaded and loaded (15 kg) conditions.

**Figure 2:**
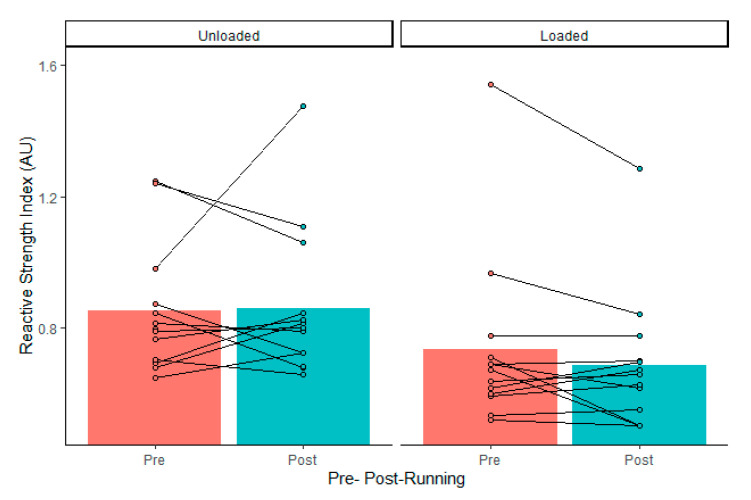
Group average (gray bars) and individual data (dots and lines) for drop jump reactive strength index before (Pre) and after (Post) the maximal graded treadmill test in unloaded and loaded (15 kg) conditions.

**Figure 3:**
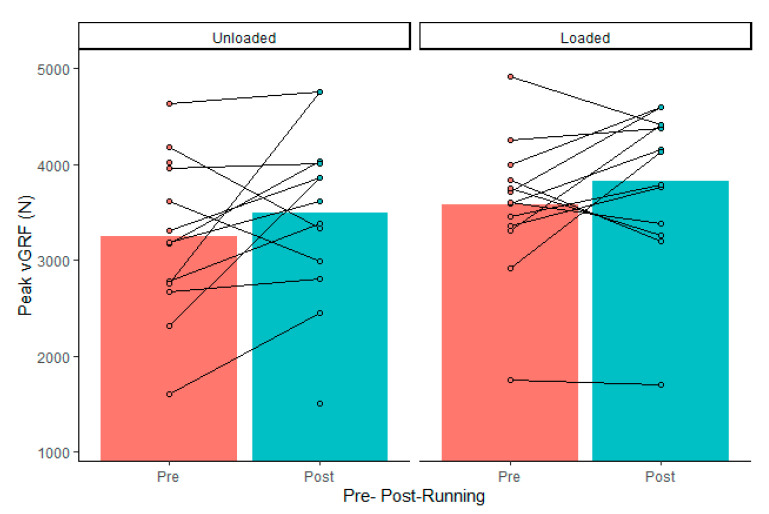
Group average (gray bars) and individual data (dots and lines) for drop jump landing peak vertical ground reaction forces (vGRF) before (Pre) and after (Post) the maximal graded treadmill test in unloaded and loaded (15 kg) conditions.

**Figure 4:**
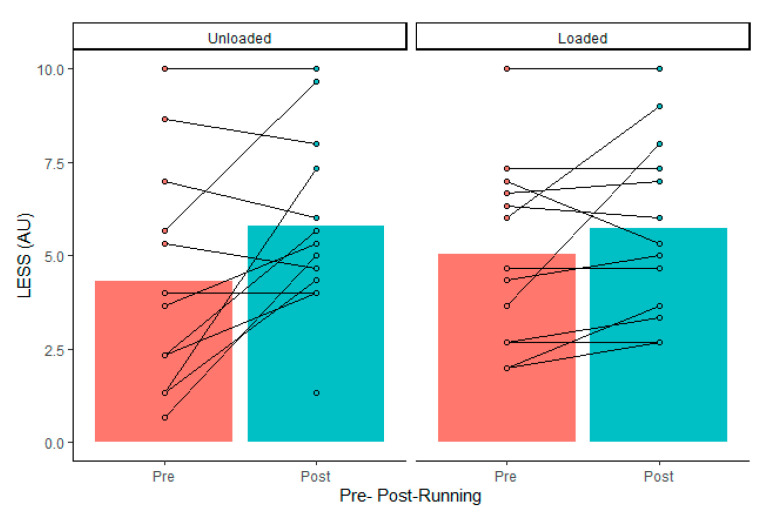
Group average (gray bars) and individual data (dots and lines) for drop jump Landing Error Scoring System (LESS) before (Pre) and after (Post) the maximal graded treadmill test in unloaded and loaded (15 kg) conditions.

**Figure 5:**
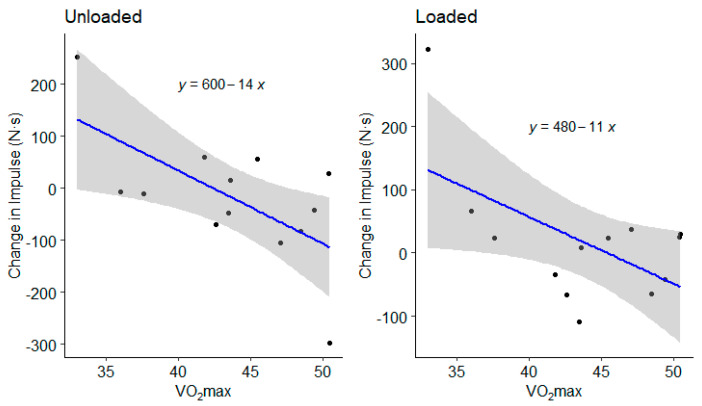
Group average (gray bars) and individual data (dots and lines) for drop jump Landing Error Scoring System (LESS) before (Pre) and after (Post) the maximal graded treadmill test in unloaded and loaded (15 kg) conditions.

Drop jump force–time metrics were considered reliable, according to coefficient of variation calculations, except for landing rate of force development (impulse, 3 ± 3%; peak cGRF, 8 ± 8%; rate of force development, 15 ± 10%; contact time, 5 ± 4%; reactive strength index, 7 ± 4%; jump height, 7 ± 7%; LESS, 5 ± 5%). There was no significant external load by fatigue interaction (*p* > 0.05). Dropping with 15 kg resulted in greater landing impulse and increased contact time, which resulted in lower jump heights and reactive strength indexes (Table 1). The maximal treadmill test did not alter drop jump vGRFs, mechanics, or performance (Table 2). There was no load by fatigue interaction (*p* > 0.05). However, 95% confidence intervals included large effects of fatigue on landing force, jump height, and LESS (Table 2). Although there were wide confidence intervals, a high percentage of individuals in the group experienced more than −10% increases (Table 3) in landing rate of force development, jump height (Figure 1), and RSI (Figure 2), as well as >10% increases in peak vGRF (Figure 3) and LESS (Figure 4). Lastly, relative VO_2max_ was associated with impulse changes from pre- to post-fatigue in unloaded (R^2^ = 0.41; *p* = 0.018) and loaded conditions (R^2^ = 0.32; *p* = 0.044) (Figure 5). Relative VO_2max_ did not predict changes in any other force–time metric due to the maximal effort bout of running.

## 4. Discussion

Body armor can protect against serious trauma, but the external loading likely reduces musculoskeletal capabilities and potentially increases injury risk [30]. The high-risk environments of MSD special agents make the body armor a necessity, subsequently creating a higher physiological demand for all movements by special agents [31]. To combat the additional physiological strain, the agents require high levels of balanced strength and aerobic capacity to withstand the external load during powerful and fatiguing movements. Furthermore, agents may require additional attention to proper movement technique, as improper movement strategies increase the effect of body armor on physiological strain [31]. Thus, it is important to consider the effects of body armor and external loading under a fatigued state, as this will likely have more real-world applications for special agents and other tactical personnel [32]. Considering the prior training and preparation of this unique group of special agents, their daily experiences may alter their responses to external loading and fatigue induced by maximal effort running, compared to previously investigated populations. Moreover, considering the uniqueness of the current group of special agents, they may require specific training needs to prepare them for future operations through improved movement competency, power output, and aerobic capacity. The current study sought to examine the effect of load and fatigue on landing forces and mechanics in Department of State MSD Special Agents.

Generally, when the mass of external loading is increased the physiological strain and decrements in movement capabilities (i.e., jump height) are exacerbated [31]. In prior research, when military personnel were equipped with heavier loads (20 and 40 kg), peak landing forces increased as the external loads became heavier [18]. To reduce the peak forces and enhance energy absorption at the knee while under load (20–40 kg), others found that military personnel relied upon more hip and knee extension when landing from 30 cm, a strategy that may reduce injury risk [33]. Yet, special agents in the current study did not alter movement mechanics according to the LESS, which is in line with prior literature examining the LESS across various loading conditions [32]. Instead, the current special agents adopted slower pacing strategies (longer total contact time) to handle the additional external load, which resulted in greater impulses but not peak vGRFs. Slower pacing strategies adopted during loaded jumping tasks have also been noted in United States Marines and led to reductions in countermovement jump heights and reactive strength indexes with a 10 kg weighted vest and a 20 kg barbell [11]. Other research in Army Reserve Officer Training Cadets also reported slower pacing strategies, but no change in joint angles (i.e., knee valgus), during drop jumps from a 30 cm box with a 15 kg weighted vest [14]. The lower jump heights and reactive strength indexes that coincided with reduced hip, knee, and ankle joint velocities and center of mass velocity, were lower in magnitude for cadets that had greater knee extensor strength [14]. Thus, the overall forces imposed on an individual during landing tasks with external loads, as well as their ability to quickly move after ground contact (i.e., jump height), may be highly influenced by contact times. Thus, tactical personnel may benefit from plyometric training aimed to explosively transition from eccentric to concentric phases to continue to improve their ability to perform landing and jumping tasks, particularly under loaded conditions.

Contrary to the original hypothesis of a greater impact of load carriage following a fatiguing protocol, there was no greater effect of external load after running to voluntary exhaustion. The aforementioned result disagrees with prior hypotheses [32] and findings of a greater impact of external loading on peak vGRFs following a bout of intense running [18]. Furthermore, the current results indicated that short bouts of running to momentary volitional fatigue did not impact landing vGRFs or movement mechanics (LESS). Others have found that an intense run prior to performing drop jumps did not influence performance under unloaded conditions, but did decrease jump height by 6% in loaded conditions (7.65 ± 0.73 kg) which were 12% lower than unloaded conditions at baseline [19]. Despite the lack of significance in the current study, the 15 kg loaded condition was similarly impacted by running induced fatigue with a 10% decline in jump height compared to 7% in the unloaded condition. In other populations, jump height and landing peak vGRFs were not impacted by running induced fatigue, but knee valgus increased [24]. Notably, and possibly driven by greater knee valgus, prior literature has found fatigued states to result in higher LESS scores [16]. Yet, the current study has demonstrated a wide range of LESS results following maximal treadmill running. This may partially be explained by various intents of jumping, despite instructions to jump as high as possible for every attempt. Resultantly, some may have moved more cautiously with lower jump height performance, while others may have maintained explosiveness at the cost of impaired movement patterns. Another potential explanation for the discrepancies in findings, is that jumping performance may be more influenced by jumping induced fatigue than running induced fatigue [34]. Thus, more research may be warranted to investigate motor control and performance after various fatiguing events experienced by tactical populations (such as obstacle course trainings involving a combination of movements).

Additionally, individuals with a greater relative VO_2max_ had less of an increase in landing impulse due to the maximal treadmill test. Thus, those with greater aerobic capacity may be less affected by fatigue in unloaded and loaded conditions. This is an important finding as the ability to resist fatigue may be associated with lower risk of lower extremity knee injuries, according to a recent systematic review [35]. With the current findings, it is encouraged that aerobic capacity be trained in conjunction with plyometric training with 15 kg or less to reduce the impact of load carriage and fatigue on landing mechanics and vGRFs. However, despite adequate power, the sample size in the current study was still relatively small and the resultant findings should therefore be considered with caution. Still the findings expand on prior literature and should be used to direct future training studies aimed at improving the efficiency of strength and conditioning program design in tactical populations.

In summary, landing while carrying external loads of 15 kg may result in slower pacing strategies (i.e., prolonged ground contact) that increase landing vGRF impulse and reduce performance capabilities (i.e., jump height and reactive strength). Short bouts of movement to volitional fatigue may not severely impact landing forces or mechanics (LESS) for the entire group, but improvements in aerobic capacities reduces the influence of fatigue on landing impulses. These findings should be considered when implementing physical training protocols for tactical populations that perform occupational tasks under external loads or fatigued states. The current findings suggest that a concomitant training approach towards improvements on reactive strength and lower body power, as well as aerobic capacity, is necessary for tactical personnel. It is also noteworthy that force–time characteristics and jump performance, although not significantly large, may be altered if exercise is conducted prior to force plate testing. Yet, these preliminary findings should be supported by future interventions.

## Figures and Tables

**Table 1:** The result of load on drop jump forces and performance.

	Unloaded	Loaded	Effect Size (CI 95%)
Impulse (N∙s)	1043.4 ± 155.3	1242.9 ± 200.0	1.115 ± 0.298 (0.51, 1.68) *
Peak vGRF (N)	3369.4 ± 863.5	3702.8 ± 766.8	0.408 ± 0.280 (−0.15, 0.95)
RFD (N∙s^−1^)	4191.3 ± 1384.7	4171.7 ± 1758.1	0.012 ± 0.277 (−0.53, 0.56)
Contact Time (s)	0.638 ± 0.105	0.703 ± 0.124	0.563 ± 0.283 (0.00, 1.11) *
RSI (AU)	0.858 ± 0.209	0.712 ± 0.236	0.653 ± 0.284 (0.09, 1.20) *
Jump Height (cm)	34.96 ± 8.24	28.40 ± 7.24	0.846 ± 0.289 (0.27, 1.40) *
LESS (AU)	5.05 ± 2.73	5.38 ± 2.40	0.130 ± 0.278 (−0.42, 0.67)

Values are mean ± standard deviation for unloaded and loaded (15 kg) conditions. For effect size, values are Cohen’s D effect size ± standard error of the effect size estimate (95% confidence intervals, CI 95%). *, indicates statistical significance. vGRF, vertical ground reaction force; RFD, rate of force development; RSI, reactive strength index; LESS, landing error scoring system, total score; AU, arbitrary units.

**Table 2:** The result of fatigue on drop jump forces and performance.

	Pre	Post	Effect Size (CI 95%)
Impulse (N∙s)	1143.7 ± 198.5	1142.6 ± 213.1	0.006 ± 0.277 (−0.54, 0.55)
Peak vGRF (N)	3411.2 ± 789.5	3660.9 ± 857.7	0.303 ± 0.279 (−0.25, 0.85)
RFD (N∙s^−1^)	4416.9 ± 1412.1	3946.2 ± 1702.7	0.301 ± 0.279 (−0.25, 0.84)
Contact Time (s)	0.675 ± 0.117	0.667 ± 0.122	0.065 ± 0.277 (−0.48, 0.61)
RSI (AU)	0.794 ± 0.238	0.775 ± 0.233	0.081 ± 0.277 (−0.46, 0.62)
Jump Height (cm)	33.53 ± 9.45	29.84 ± 6.79	0.449 ± 0.281 (−0.11, 0.99)
LESS (AU)	4.67 ± 2.65	5.77 ± 2.36	0.439 ± 0.281 (−0.12, 0.98)

Values are mean ± standard deviation for unloaded and loaded (15 kg) conditions. For effect size, values are Cohen’s D effect size ± standard error of the effect size estimate (95% confidence intervals, CI 95%). Indicates statistical significance. vGRF, vertical ground reaction force; RFD, rate of force development; RSI, reactive strength index; LESS, landing error scoring system, total score.

**Table 3:** Number of participants experiencing >10% change in performance due to maximal treadmill running.

Variable	Change	Unloaded # (%)	Loaded # (%)
Impulse	Decrease	1 (8)	1 (8)
Peak vGRF	Increase	6 (46)	6 (46)
RFD	Decrease	7 (54)	7 (54)
Contact Time	Increase	1 (8)	2 (15)
RSI	Decrease	4 (31)	5 (38)
Jump Height	Decrease	3 (23)	4 (31)
LESS	Increase	7 (54)	6 (46)

#, number of agents with corresponding percent change out of the 13 total sample. vGRF, vertical ground reaction force; RFD, rate of force development; RSI, reactive strength index; LESS, landing error scoring system, total score.

## Data Availability

The data presented in this study are available on request from the corresponding author.
